# Optimising child-friendly TB preventive treatment regimens

**DOI:** 10.5588/ijtldopen.25.0369

**Published:** 2025-11-12

**Authors:** C. Mulder, A.J. Bergman, F. Mulatu, R. Chaisson, G. Churchyard, A. Bedru, D. Kerrigan, N. Salazar-Austin

**Affiliations:** 1 Department of TB Elimination and Health System Innovations, KNCV Tuberculosis Foundation, The Hague, the Netherlands;; 2 Amsterdam Institute for Global Health and Development, Amsterdam University Medical Centres, Amsterdam, the Netherlands;; 3 University of Virginia School of Nursing, Charlottesville, VA, USA;; 4 KNCV Ethiopia, Addis Ababa, Ethiopia;; 5 Department of Medicine, Johns Hopkins University School of Medicine, Baltimore, MD, USA;; 6 The Aurum Institute, Johannesburg, South Africa;; 7 Department of Preventive Medicine, George Washington School of Public Health, Washington, DC, USA;; 8 Department of Pediatrics, Johns Hopkins University School of Medicine, Baltimore, MD, USA.

**Keywords:** TB, Ethiopia, short-course regimens, paediatric TB, caregivers, isoniazid, rifapentine, rifampicin

## Abstract

**BACKGROUND:**

The World Health Organization recommends rifamycin-based TB preventive treatment (TPT) for children, but factors influencing regimen acceptability are not well understood. We explored perceptions of two short-course regimens – 3HP (weekly dosing) and 3RH (daily dosing) – among interest-holders in Ethiopia.

**METHODS:**

Forty-seven in-depth interviews were conducted with caregivers of TB-exposed children, community health workers, health care providers, and policymakers following the CHIP-TB trial, which evaluated home-based TPT delivery. Thematic content analysis was used to assess acceptability across factors such as palatability, dosing frequency, pill burden, adherence, and side effects.

**RESULTS:**

Both regimens were generally well tolerated. Caregivers reported good palatability, though some children vomited or spit out the medication. Weekly dosing with 3HP was seen as less burdensome but easier to forget, requiring additional support for adherence. Daily 3RH was considered more difficult due to the routine burden on caregivers. Interest-holders preferred 3HP overall but noted concerns about the high number of pills per dose. Mild side effects were reported with both regimens but did not result in discontinuation.

**CONCLUSION:**

Interest-holders favoured 3HP for its lower dosing burden, despite concerns about pill burden. Child-friendly, fixed-dose formulations and consideration of caregiver and child preferences are essential to improving adherence and treatment outcomes.

TB remains a global public health challenge, particularly in low-resource settings such as Ethiopia.^1^ Each year, an estimated 1 million children <15 years of age fall ill with TB.^1^ TB preventive treatment (TPT) is highly effective in preventing the progression to TB disease and is recommended by the World Health Organization (WHO) for all close child-TB contacts <15 years of age.^2–4^ The WHO recommends short-course TPT regimens including 3HP (12 weekly doses of isoniazid and rifapentine)^2,5^ and 3RH (daily dose of rifampicin and isoniazid for 3 months). Paediatric implementation of 3HP has been hindered by the lack of child-friendly formulations, high pill burden, absence of dosing for children <2 years, and limited data on the drug–drug interaction between rifapentine and dolutegravir.^6^ 3RH is widely used and administered as a dispersible, fixed-dosed combination.^2,7^ Previous discrete choice experiments showed a preference for daily over weekly medication for improved adherence.^8,9^ Qualitative in-depth interviews among parent–child dyads having experience with both 3HP and 6H (daily dose of isoniazid for 6 months) showed a preference for dispersible 3HP, but participants remained concerned about its ability to be integrated into a weekly routine.^10^

To bolster paediatric TPT uptake and adherence in Ethiopia, it is essential to understand interest-holders’ perspectives comparing 3HP and 3RH. We explored the experiences of caregivers, community health workers (CHWs), health care providers, and programme managers with both the 3HP and 3RH regimens given to children in a nested, qualitative sub-study of the CHIP-TB trial in Ethiopia.

## METHODS

Individual, in-depth interviews were conducted among interest-holders following the CHIP-TB trial, a pragmatic, cluster-randomised trial designed to evaluate home-based TPT services delivered by CHWs versus the facility-based standard of care.^11–14^ Interviews explored interest-holders’ experiences with the intervention and the TPT regimens; thus, only interest-holders involved with the home-based intervention were interviewed.

### Study setting

Interviews were conducted from October 2022 to February 2023 in the East Shewa Zone of Oromia, Ethiopia. According to national guidelines, infants and children <2 years of age were prescribed 3RH, and children ≥2 years of age were prescribed 3HP as first choice but could alternatively have been prescribed 3RH, when preferred by caregivers or when 3HP was unavailable.^15^ 3RH was a fixed-dose combination and dispersible tablet, whereas 3HP was separate, film-coated tablets that required crushing for children who could not swallow whole tablets.

### Study population

Participants were purposively selected to ensure diverse representation of consumer, clinical, and programmatic perspectives. We attempted to vary gender, age, and health centre to capture a wide range of perspectives. Programme managers included TB and Community Health Program coordinators at the local and regional levels. Caregivers were selected to vary experience, including caregivers with older (6–15 years) versus younger children (≤5 years), those whose children had experience with both regimens, and those who were and were not the person being treated for TB themselves. Eligible providers included TB nurses and public health providers. Providers, CHWs, and caregivers were all recruited from the health centres implementing the CHIP-TB intervention.

### Procedures

Semi-structured field guides were developed to explore interest-holders’ perceptions of the intervention and TPT regimens. The interviews, lasting approximately 1 h, were conducted in Afan Oromo or Amharic by local research assistants trained in qualitative methods who were not involved in the trial. All interviews were audio-recorded, transcribed, de-identified, and translated into English for analysis. Transcripts were reviewed for accuracy, and the analytic team collaborated with the translation team and local study coordinator to clarify ambiguities and provide context. We temporarily paused data collection after the first two interviews to conduct a data quality assessment and did not identify any significant limitations.

### Analysis

Transcripts were analysed using thematic content analysis using both deductive and inductive approaches. A priori codes were developed based on the implementation-focused aims of the study and interview guides developed from the study team’s experience during the trial. Emergent codes were developed as unexpected domains were identified during the coding process. Limited emergent codes were identified in the data following the initial 10–12 caregiver and CHW interview transcripts indicating meaningful saturation.^16^ Data coding and analysis were performed by two team members trained in qualitative research methods. Coding was conducted using ATLAS.ti© software. Researchers met weekly to discuss data interpretation and explore emergent codes. Revisions to the codebook were iterative throughout the coding process. We defined regimen acceptability as it was influenced by palatability, ease of ingestion, pill burden, dosing frequency, and adverse effects, which in turn influence adherence and completion. We did not rely on data from all interest-holders for every attribute, as their proximity to the relevant information varied.

### Ethical statement

Programme managers, providers, and CHWs were approached directly by study staff, while caregivers were recruited by health centre staff. Interviews were conducted in private locations chosen by the participants. Written informed consent was obtained after participants were adequately informed, and they were compensated for their time. The study protocol was reviewed and approved by the Oromia Regional Health Bureau Public Emergency and Health Research Directorate Institutional Review Board (BEFO/HBTFH/1-16/10200/18/08/2013), the Johns Hopkins Medicine Institutional Review Board (IRB00237243), and the World Health Organization’s Ethics Review Committee (ERC.0003244).

## RESULTS

A total of 47 in-depth interviews were conducted, including 15 caregivers of children exposed to TB, 15 CHWs, 9 TB providers, and 8 programme managers. Of the 15 caregivers, 5 had experience with providing 3HP to their children, 9 providing 3RH, and 1 with both 3HP and 3RH (Table 1). All CHWs were female, whereas the providers and programme managers were predominantly male (Table 2).

**Table 1.** Caregiver characteristics.

	Caregiver (n = 15)
Sex	
Female	12 (80%)
Male	3 (20%)
Age, median (IQR)	34 (26, 38)
TPT regimen prescribed	
3HP	5 (33%)
3RH	9 (60%)
3HP and 3RH	1 (7%)
Relationship to child	
Parent	13 (87%)
Grandparent	1 (7%)
Sibling	1 (7%)

**Table 2.** Characteristics of community health workers, TB providers, and programme managers.

	Community health workers (n = 15)	TB providers (n = 9)	Programme managers (n = 8)
Sex			
Female	15 (100%)	0 (0%)	1 (13%)
Male	0 (0%)	9 (100%)	7 (88%)
Age, median (IQR)	28 (26, 30)	32 (32, 35)	41 (30, 47)
Years of experience in current role			
<5 years	5 (33%)	1 (11%)	5 (63%)
5–10 years	3 (20%)	7 (78%)	2 (25%)
>10 years	7 (47%)	1 (11%)	1 (13%)

### Palatability and ease of ingestion

Caregivers reported 3HP to be similarly palatable to 3RH; caregivers of children on both regimens described how their children vomited or wanted to spit out the medication.

When they [my 4-year-old] chew, its taste is another thing, when he chews it changes, that was why he swallow half and drop out half of it.

(Female, parent to 4- and 9-year-old children on 3HP)

Palatability influenced caregiver dosing preferences. This same caregiver preferred a weekly regimen.

The weekly one is better because if you provide the drug to a child on daily basis, he/she hesitates to take the drug.

Some caregivers explicitly mentioned the challenges of administering TPT, stating that they sometimes had to force their children to take the medication.

We make younger children swallow the drug by making faces on them, the older one by telling them it is for health, they swallow it to be safe from it.

(Male, parent to five children <15 years on 3HP)

Administering TPT to older children was reported to be easier, and for younger children caregivers noted that dissolving the drugs in water, tea, or breast milk, regardless of the regimen, facilitated administration.

I dissolved in a teacup and I provided him with tea but he vomit it. Then when I dissolved in soup the colour changed. I thought he may not take. I started to mix with food in the third day and the child started to take it. I crush the drug into two pieces and I provided him both pieces by mixing with food. That was how I finished the drug.

(Female, parent of 2- and 7-year-old children on 3RH. Quote refers to 2-year-old)

### Dosing frequency and pill burden

Caregivers valued a weekly dosing schedule as it reduced overall burden for the child.

I preferred the weekly one because the child does not hesitate to take it if given on a weekly basis. However, if you give them the drug every day children may hate it and refuse to take it.

(Female, parent to 4- and 9-year-old children on 3HP)

Moreover, daily dosing was found to be burdensome as it was not perceived to allow flexibility in the timing of administration.

Yes, it was difficult to make children swallow this drug. For instance, if I were providing at one o’clock the time must be fixed, you cannot provide before one o’clock or after one o’clock.

(Male, parent to 5-year-old and caregiver to 8- and 12-year-old children on 3RH)

While not a view shared by caregivers, CHWs expressed some concern about the large number of pills required per 3HP dose.

In my opinion, I think swallowing many drugs at a time might be more challenging to children than taking one every day, it may be more harmful than the daily dose. I think families and children might choose the weekly dose because they consider frequency but the burden of drugs or the number of drugs are a challenge. I think the family prefers the weekly dose because the days are fewer, but I prefer the daily dose.

(CHW, 5–10 years’ work experience)

Programme managers and providers generally demonstrated greater acceptance and preference for prescribing 3HP over 3RH; however, the higher number of pills per dose in the 3HP regimen was cited as a disadvantage, and the need for a fixed-dose, child-friendly formulation was emphasised by one provider.

If the drugs are combined, it means that there is a difference between swallowing 10 pills and 5 pills. It means that it also affects the family that receives it. I think if it is supplied in combined form, it would make the job easier.

(TB provider, <5 years’ work experience)

### Adherence

All interest-holder groups acknowledged that weekly doses could be difficult to remember, but there were also messaging and implementation strategies utilised that could help support adherence. Dosing was often provided on the weekend for two reasons. First, this would avoid providing medication on a school day, allowing children to avoid potential side effects during school and for parents to schedule around the older children’s busy school schedule.

Because these children are students and I may not provide to them on the exact time. I may also forget to provide the drug. It is not comfortable for me if it would be given daily because I am a student and the children also stay in school till 3 PM. Since the weekly one is given on Saturday I’m happy with the weekly drug.

(Sibling, female, age 19, child on 3HP)

Second, CHWs used individualised adherence counselling to select the appropriate day of the week, which often meant scheduling it around that family’s religious practices.

Before starting the drug, we ask them, ‘What day of the week is convenient for you? What day is best for you to give the drug to your child?’ If they are Muslims, it is not convenient on Friday, and it could be on another day. However, it is convenient on the other day for non-Muslim clients, and we give them based on that.

(CHW, <5 years’ work experience)

Some CHWs conducted weekly adherence visits, which provided an opportunity for close monitoring and counselling.

3HP was given per week. The simple thing is that we deliver it weekly. Due to this, there’s even the moment you sit there and they take it. I think that one is good. But they took 3RH daily. That can create a gap because we are not available to them.

(CHW, 5–10 years’ work experience)

Adherence counselling should be strengthened to prevent confusion between TPT and TB treatment.

A father of one child said when he started taking RH, he said, why is the little child taking the drug I am taking? He said to me. This approach created a problem and effects, it is good if changed or corrected, and it would be good if replaced by the weekly dose drug.

(HEW, age 32, >10 years’ work experience)

### Side effects and tolerability

Across both regimens, interest-holders reported generally mild side effects which did not result in treatment discontinuation. Some caregivers of children on the 3RH regimen reported that discolouration of the urine caused their children distress.

It changed their colour of urine to red. That was why they were scared. But health extension told us about this colour change.

(Male, parent to 5-year-old and caregiver to 8- and 12-year-old children on 3RH)

## DISCUSSION

This qualitative analysis highlighted diverse perspectives on key attributes of 3HP and 3RH in children. Regarding palatability, a minority of caregivers expressed concerns about the taste and odour of the medications, while CHWs and health care providers generally had positive views of both regimens. Dissolving TPT in water or breast milk facilitated adherence, and ease of administration seemed to improve with age. Dosing frequency was crucial to acceptability, all interest-holders, and in particular caregivers, favoured the 12-dose 3HP regimen over the 90-dose 3RH regimen. All interest-holder groups agreed that treatment completion was achievable with 3HP and 3RH. Nevertheless, the dosing schedules elicited mixed responses, with weekly doses viewed as easier to forget but daily dosing considered burdensome and difficult to manage. Our findings highlight that TPT uptake and completion might be most successful if the TPT regimen is well matched to the family’s preference.

Our findings align with a qualitative study from Lesotho, which identified pill burden, treatment duration, and dosing frequency as key factors influencing caregivers’ preferences for TPT, with caregivers expressing a preference for shorter treatment periods and less-frequent drug administration.^17^ In contrast, our results differ from caregivers in Eswatini, suggesting that among caregivers, dosing frequency may play a more significant role in influencing treatment adherence than the specific drug formulations used.^8^ Earlier studies, including recent paediatric research, show a clear preference for weekly dosing due to minimal drug administration burden, though concerns about adherence to such a dosing schedule remain.^9,10^

The dispersible rifapentine formulation, which became available in 2024, combined with the dispersible isoniazid formulation, is expected to improve 3HP administration.^18^ A qualitative sub-study in South Africa demonstrated that most children ingested 3HP with limited difficulty, highlighting the practicality and acceptability of the dispersible rifapentine formulation for paediatric use.^10^ In a qualitative study from Peru, caregivers reported having a child-friendly formulation – such as dispersible tablets or syrups – as the most important factor in a TPT regimen.^19^ However, our data indicate there remains a need for a combined dispersible formulation in a fixed-dose to reduce the 3HP pill burden.

In our data, CHWs noted that the distinctiveness of the 3HP regimen from standard TB treatment served as an influential factor for caregivers to initiate TPT, as it was perceived as a separate intervention for their healthy, TB-exposed children. This highlights the importance of counselling to reinforce the preventive nature of TPT. Regimens that clearly differentiate TPT from TB treatment may further support uptake and adherence. Ease of adherence, determined by an amalgam of drug administration factors, is crucial when developing future TPT regimens for children. A recent modelling study conducted among household contacts of all ages across a wide range of country settings identified ease of adherence as the second strongest predictor of the epidemiological impact of TPT regimens, following regimen efficacy.^20^ This highlights the importance of prioritising child-friendly drug attributes, particularly since regimen efficacy is comparable across rifamycin-based TPT regimens.^2^

This qualitative study was conducted following a larger trial, allowing for a rigorous sampling and analytical approach. A purposive sampling frame helped to ensure varied and rich data. Furthermore, the data were triangulated across various interest-holder groups, providing a comprehensive perspective within a high-TB-burden setting. This approach strengthens the reliability and rigour of our findings. A limitation of this study was that during the trial, we experienced a stockout of 3HP, which resulted in all child contacts receiving 3RH during that period. This stockout reflects the programmatic reality many health care systems face. However, health care workers gained experience with both regimens in children aged 2–15 years. This study benefited from CHW support and structured implementation strategies that likely enhanced adherence; however, such support may not be available in all settings, and programmes should consider local capacity when planning TPT implementation.

## CONCLUSION

Our study highlighted that dosing frequency is a critical factor influencing the acceptability of TPT regimens in children. In this sample, interest-holders generally favoured the 12-dose 3HP regimen over the 90-dose 3RH regimen due to its reduced dosing burden although perspectives varied. While qualitative data are not intended to be generalisable, these findings may be transferrable to caregivers and families in similar circumstances who are balancing dynamic family needs and schedules. To accelerate the uptake of TPT and improve programmatic management, it is essential to prioritise counselling that aligns regimens with family preferences. There is an urgent need to accelerate the development and adoption of person-centred, highly acceptable, and child-friendly formulations. These efforts are crucial to ensuring that TPT regimens meet the needs of children and their caregivers, ultimately enhancing adherence and programme impact.
